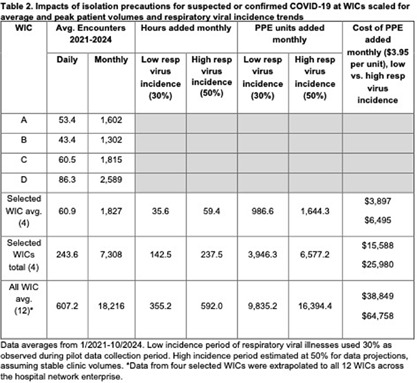# The Unintended Burden of the Use of Transmission-Based Precautions for Suspected COVID-19 Patients in the Ambulatory Setting

**DOI:** 10.1017/ash.2025.298

**Published:** 2025-09-24

**Authors:** Rebecca Stern, Tom Talbot, Katherine Bashaw

**Affiliations:** 1 Vanderbilt University Medical Center; 2 Vanderbilt University School of Medicine; 3 VUMC

## Abstract

**Background:** Implementation of transmission-based precautions has predominantly been performed in inpatient acute care settings. Limited guidance is available on applying these precautions in ambulatory clinics, especially for patients with suspected or confirmed COVID-19. This timed analysis of empiric isolation precautions for COVID-19 in walk-in clinics (WIC) aimed to identify unintended impacts that are underappreciated with inpatient use. **Methods:** An observational analysis at four WIC sites in an academic hospital network was conducted in July-October 2024. Patients who screened positive at check-in with cough, sore throat, congestion, or recent COVID-19 positive testing triggered an electronic notification on the need for airborne and contact isolation precautions with eye protection. A timed evaluation of healthcare personnel (HCP) to don and doff personal protective equipment (PPE) upon patient room entry and exit was performed by two observers using a standardized process with a stopwatch. HCP were surveyed regarding attitudes and barriers using a 5-point Likert scale on REDCap. **Results:** Sixty patient encounters requiring COVID-19 isolation were observed, representing 30.4% of the total WIC patients seen during the observation periods (N=197 over 36.5 hours). Cough and sore throat were the most common symptoms triggering isolation (both 55%). The mean time to don and doff PPE per room entry and exit was 1.58 and 0.57 minutes, respectively (2.16 minutes per don and doff cycle; Table 1). HCP performed donning and doffing an average of 1.8 times (range 1-4) per patient. Extrapolated to a 12-hour shift, this adds 1.3 hours to daily activities and encompasses 35 sets of PPE (e.g. gowns, gloves, eye protection, respirators), contributing to WIC waste volumes (Table 2). HCP survey respondents (N=26/49) indicated a majority strong agreement that PPE increased the time required, burden to HCP, and waste. **Conclusions:** Multiple workflow, resource, and HCP burdens of using full COVID-19 isolation precautions for WIC patients suggest that refining isolation criteria for ambulatory settings may help preserve clinic efficiency and limit waste. This pilot occurred during a period with low COVID-19 and influenza-like illness incidence, underscoring the challenges of scaling empiric transmission-based precautions to high-volume clinics during surges of respiratory virus season. Further studies are needed to evaluate the impacts of eliminating the gown and gloves components of PPE for COVID-19 in ambulatory settings, which may be unnecessary given the lower likelihood of transmission by non-airborne routes, short duration of outpatient clinic encounters which limits environmental contamination with SARS-CoV-2 virus, and lack of aerosol-generating procedures.

**Figure:**
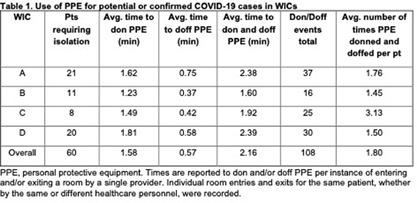

**Figure:**